# Safety and Efficacy of Half-Half Inflation Strategy in Pulmonary Artery Stenosis Caused by Fibrosing Mediastinitis

**DOI:** 10.1016/j.jacasi.2025.03.005

**Published:** 2025-05-13

**Authors:** Hongling Su, Yaling Chen, Aqian Wang, Kaiyu Jiang, Yating Zhao, Mengfei Jia, Bo Li, Xuechun Sun, Wenjie Dong, Yunshan Cao

**Affiliations:** a Department of Cardiology, Pulmonary Vascular Disease Center, Gansu Provincial Hospital, Lanzhou, China; b Department of Cardiology, Gansu Provincial Central Hospital, Lanzhou, China; c School of Medicine, Jiangsu University, Zhenjiang, China; d The Second Clinical Medical School, Lanzhou University, Lanzhou, China; e Heart, Lung and Vessels Center, Sichuan Provincial People's Hospital, University of Electronic Science and Technology of China, Chengdu, China; f Department of Cardiology, The First People’s Hospital of Tianshui, Tianshui, China

**Keywords:** fibrosing mediastinitis, percutaneous transluminal pulmonary angioplasty, pulmonary artery stenosis, pulmonary hypertension

Fibrosing mediastinitis (FM) is a rare fibroproliferative disease characterized by the proliferation of fibrous tissue that compresses mediastinal structures, including pulmonary arteries (PAs), potentially leading to pulmonary hypertension (PH) and poor survival.^1^ Percutaneous transluminal pulmonary angioplasty (PTPA) has shown promise in treating PA stenosis; however, its effectiveness in FM-associated pulmonary artery stenosis (FM-PAS) remains unclear.^1^ We evaluated the safety and efficacy of PTPA using different balloon inflation strategies in FM-PAS.

This study retrospectively enrolled FM-PAS patients who underwent PTPA at Gansu Provincial Hospital between April 2018 and June 2022. Patients aged <18 or >80 years were excluded. The study protocol (2022-302) was approved by the Institutional Ethics Committee with an informed consent waiver. Clinical data were collected. Translesional pressure gradients were measured, with PTPA indicated for gradients exceeding 10 mm Hg. Reference vessel diameter was calculated as the average of proximal and distal reference diameters. Acute gain was measured as the difference in minimum lumen diameter preintervention and postintervention.

Two balloon inflation strategies were employed: rapid inflation (the traditional strategy) and gradual inflation (the "half-half" strategy). The traditional strategy involved directly inflating the balloon to a nominated pressure (usually 6 atmospheres) initially, with subsequent adjustments based on angiographic results or complications. Conversely, the “half-half” strategy started with an inflation to one-half of the nominated pressure (usually 3 atmospheres), followed by gradual 0.5-atmosphere increments every 5 to 10 seconds. Inflation was halted and deflated gradually if patients experienced discomfort (coughing, chest pain) and resumed only after full symptom relief and vital sign stabilization. If cough and other discomfort occurred repeatedly, the doctor decided whether to continue to inflate according to the risk of hemoptysis. If hemoptysis occurred, even minor hemoptysis, the procedure was discontinued. Stent implantation was reserved for cases where balloon angioplasty alone proved insufficient caused by elastic recoil. In the “half-half” strategy group, incomplete stent expansion was deemed acceptable, with planned staged postdilation in subsequent sessions if necessary. In-stent restenosis (ISR) was defined as minimum lumen diameter within or adjacent to the stent <50% of the original stent diameter, or a translesional pressure gradient increase ≥10 mm Hg. Reperfusion pulmonary injury (RPI) was defined as a decrease in oxygen saturation occurring during or within 24 hours after the intervention. Severe complications included massive hemoptysis, severe RPI, PA rupture, hemothorax, stent displacement, and death. Postprocedure, dual antiplatelet therapy (aspirin 100 mg/d, clopidogrel 75 mg/d) was administered for 1 to 6 months, followed by single antiplatelet therapy for up to 3 to 12 months.

Data analysis was performed using SPSS version 21.0. For paired continuous variables, paired Student's *t*-tests or Wilcoxon tests were used; categorical variable was analyzed using chi-square or Fisher exact tests, with significance at *P <* 0.05.

Among 98 enrolled patients who underwent 141 PTPA sessions, the mean age was 65.8 ± 6.8 years, with a female predominance (69 of 98, 70.4%). The common comorbidities were PH (95 of 98, 96.9%), a history of tuberculosis (63 of 98, 64.3%), and COPD (54 of 98, 55.1%). The lower lobar PAs were mostly compressed (Figure 1A). At a median follow-up of 8.5 months (Q1-Q3: 5-15 months), significant improvements were observed in exercise capacity and hemodynamic parameters (Figure 1B).

**Figure 1:**
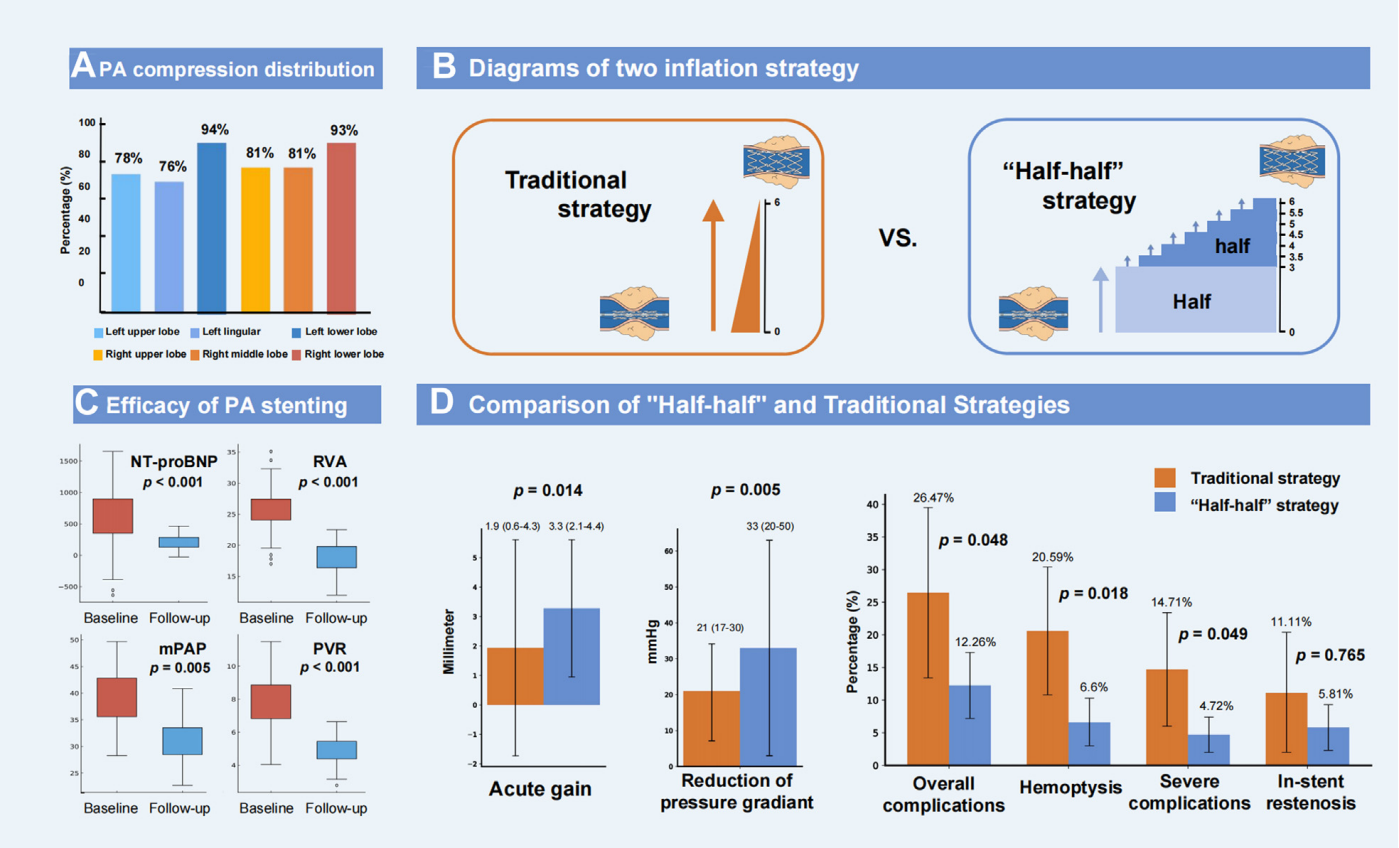
Safety and Efficacy of Half-Half Inflation in FM-PAS (A) Pulmonary artery (PA) compression distribution. (B) Diagrams of 2 inflation strategy. (C) Efficacy of PA stenting. (D) Comparison of "half-half" and traditional strategies. mPAP = mean pulmonary artery pressure; NT-proBNP = N-terminal pro–B-type natriuretic peptide; PVR = pulmonary vessel resistance; RVA = right ventricular area.

Patients were stratified into the traditional strategy group (20 patients, 36 PAs in 23 sessions) and the “half-half” group (72 patients, 136 PAs in 89 sessions). Baseline characteristics showed no significant differences between the 2 groups. Compared to the traditional group, the “half-half” group demonstrated a larger reference vessel diameter (9.5 vs 8.2 mm; *P =* 0.021), longer lesion length (20.9 vs 17.4 mm; *P =* 0.024), and lower balloon and stent inflation pressures (*P <* 0.001). Both strategies showed comparable improvements in exercise capacity at follow-up. The “half-half” strategy achieved superior luminal gain and greater pressure gradient reduction. Both strategies demonstrated similar RPI rates (1 of 34, 2.94% vs 4 of 106, 3.81%; *P =* 1.000). The “half-half” strategy showed lower overall complications rates (9 of 34, 12.26% vs 13 of 106, 26.47%; *P =* 0.048), and intraoperative deaths (0 of 72, 0% vs 2 of 20, 10%; *P =* 0.045). At follow-up, both groups showed similar ISR rates (Figure 1C).

This study represents the largest cohort to date evaluating the impact of PTPA on FM-PAS. The study population was notably older than the Western cohorts, suggesting possible differences in etiology.^1^ Unlike earlier studies reporting PA involvement in approximately 42% of cases,^2^ our study demonstrated predominantly bilateral PA compression with a higher percentage of PH, likely reflecting referral bias to our PH center.

At follow-up, patients exhibited significant improvements in hemodynamics, exercise capacity, and right heart remodeling. However, the overall complication rate was 15.6% (severe complication rate: 8.6%), with traditional strategy showing a higher rate (26%) consistent with previous study.^3^

Hemoptysis was the most frequent complication, likely caused by FM pathology which involves fibrous tissue invasion into vessel adventitia and bronchial cartilage,^1^ making them prone to injury during dilation. Additionally, the extensively dilated compensatory collateral branches, located within the fibrous tissue that surrounds the narrowed vessels, may be an important but often overlooked cause of hemoptysis. Therefore, the traditional strategy could not be suitable for FM-PAS. Drawing from extensive experience in coronary interventions,^4^ we adopted a gradual balloon inflation approach (the “half-half” strategy) in subsequent procedures. Fortunately, our findings supported the use of this strategy, which minimized risks while achieving comparable efficacy to the traditional approach. The reason why the immediate effect of the traditional group is slightly worse than that of the half-half group may be limited by complications, and the obvious longer inflation time of the half-half group may also be the reason why the immediate effect of the “half-half” group is better. Notably, no significant increase in ISR was observed. Previous reports showed ISR rates of 22% to 71%, which may be related to longer follow-up.^5^^,^^6^ Thus, patients with FM-PAS are more suitable for a longer period of gradual inflation. In addition, lung injury after stenting is an important issue needing to be addressed in the future.

This retrospective, single-center study requires validation through prospective trials with longer follow-ups. Additionally, the impact of pulmonary vein interventions on outcomes cannot be overlooked.

## Funding Support and Author Disclosures

This work was supported by the National Natural Science Foundation of China (No. 82070052), the Joint Fund of Science and Technology Department of Gansu Province (23JRRA1544), and the Hospital fund of Gansu Provincial Hospital (24JRRE012). The authors have reported that they have no relationships relevant to the contents of this paper to disclose.
